# Piperazine-1,4-diium bis­(hexa­hydroxido­hepta­oxidohexa­borato-κ^3^ *O*,*O*′,*O*′′)cobaltate(II) hexa­hydrate

**DOI:** 10.1107/S1600536814007090

**Published:** 2014-04-05

**Authors:** Nabil Jamai, Mohamed Rzaigui, Samah Akriche Toumi

**Affiliations:** a Laboratoire de Chimie des Matériaux, Faculté des Sciences de Bizerte, 7021 Zarzouna Bizerte, Tunisia

## Abstract

In the title hydrate, (C_4_H_12_N_2_)[Co{B_6_O_7_(OH)_6_}_2_]·6H_2_O, both the dication and dianion are generated by crystallographic inversion symmetry. The Co^2+^ ion in the dianion adopts a fairly regular CoO_6_ octa­hedral coordination geometry arising from the two *O*,*O*′,*O*′′-tridentate ligands. In the crystal, the dianions and water mol­ecules are linked by O—H⋯O hydrogen bonds, generating a framework with large [100] channels, which are occupied by the organic dications. N—H⋯O and C—H⋯O hydrogen bonds consolidate the structure.

## Related literature   

For related structures, see: Natarajan *et al.* (2003 ▶); Negro *et al.* (1971 ▶); Zhihong *et al.* (2005 ▶); Yue *et al.* (2003 ▶).
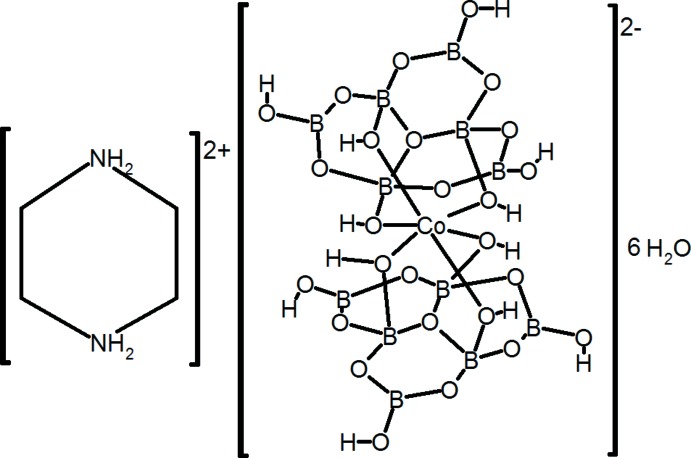


## Experimental   

### 

#### Crystal data  

(C_4_H_12_N_2_)[Co(H_6_B_6_O_13_)_2_]·6H_2_O
*M* *_r_* = 813.00
Triclinic, 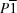

*a* = 8.226 (3) Å
*b* = 10.157 (2) Å
*c* = 11.298 (4) Å
α = 65.98 (2)°
β = 74.20 (3)°
γ = 69.03 (5)°
*V* = 796.2 (4) Å^3^
*Z* = 1
Ag *K*α radiation
λ = 0.56087 Å
μ = 0.35 mm^−1^
*T* = 298 K
0.23 × 0.15 × 0.11 mm

#### Data collection  

Enraf–Nonius CAD-4 diffractometer
9895 measured reflections
7785 independent reflections
6118 reflections with *I* > 2σ(*I*)
*R*_int_ = 0.014
2 standard reflections every 120 min intensity decay: 2%

#### Refinement  

*R*[*F* ^2^ > 2σ(*F* ^2^)] = 0.049
*wR*(*F* ^2^) = 0.153
*S* = 1.06
7785 reflections
259 parameters
12 restraints
H atoms treated by a mixture of independent and constrained refinement
Δρ_max_ = 2.42 e Å^−3^
Δρ_min_ = −0.99 e Å^−3^

### 

Data collection: *CAD-4 EXPRESS* (Enraf–Nonius, 1994 ▶); cell refinement: *CAD-4 EXPRESS*; data reduction: *XCAD4* (Harms & Wocadlo, 1996 ▶); program(s) used to solve structure: *SHELXS97* (Sheldrick, 2008 ▶); program(s) used to refine structure: *SHELXL97* (Sheldrick, 2008 ▶); molecular graphics: *ORTEP-3 for Windows* (Farrugia, 2012 ▶) and *DIAMOND* (Brandenburg & Putz, 2005 ▶); software used to prepare material for publication: *WinGX* (Farrugia, 2012 ▶).

## Supplementary Material

Crystal structure: contains datablock(s) I, global. DOI: 10.1107/S1600536814007090/hb7207sup1.cif

Structure factors: contains datablock(s) I. DOI: 10.1107/S1600536814007090/hb7207Isup2.hkl

CCDC reference: 994558

Additional supporting information:  crystallographic information; 3D view; checkCIF report

## Figures and Tables

**Table 1:** Selected bond lengths (Å)

Co1—O2	2.0539 (17)
Co1—O1	2.0648 (14)
Co1—O4	2.1926 (12)

**Table 2:** Hydrogen-bond geometry (Å, °)

*D*—H⋯*A*	*D*—H	H⋯*A*	*D*⋯*A*	*D*—H⋯*A*
O1—H1⋯O2*W*	0.82 (1)	2.00 (2)	2.7525 (19)	153 (3)
O2—H2⋯O6^i^	0.81 (1)	2.11 (1)	2.876 (2)	157 (3)
O3—H3⋯O23^ii^	0.82	1.93	2.745 (2)	172
O4—H4⋯O2*W*	0.81 (1)	2.08 (1)	2.864 (2)	164 (3)
O5—H5⋯O45^iii^	0.82	1.91	2.7159 (18)	169
O6—H6⋯O4^i^	0.82	1.95	2.734 (2)	160
O1*W*—H1*W*1⋯O5	0.86 (1)	1.96 (2)	2.771 (2)	158 (4)
O1*W*—H2*W*1⋯O26^iv^	0.86 (1)	2.27 (1)	3.108 (3)	167 (3)
O1*W*—H2*W*1⋯O23^iv^	0.86 (1)	2.51 (3)	3.158 (3)	133 (3)
O2*W*—H1*W*2⋯O3^v^	0.85 (1)	2.05 (2)	2.861 (2)	159 (3)
O2*W*—H2*W*2⋯O1*W* ^vi^	0.85 (1)	2.01 (1)	2.838 (3)	164 (3)
O3*W*—H2*W*3⋯O1*W* ^vii^	0.85 (1)	2.05 (2)	2.842 (3)	155 (3)
O3*W*—H1*W*3⋯O1^viii^	0.85 (1)	2.24 (1)	3.088 (3)	179 (1)
N1—H1*A*⋯O16	0.90	1.78	2.654 (2)	165
N1—H1*A*⋯O6	0.90	2.46	2.986 (3)	117
N1—H1*B*⋯O3*W*	0.90	1.98	2.836 (3)	157
C1—H1*C*⋯O34^v^	0.97	2.56	3.321 (3)	136

Symmetry codes: (i) 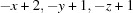; (ii) 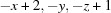; (iii) 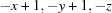; (iv) 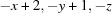; (v) 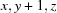; (vi) 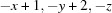; (vii) 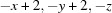; (viii) 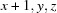.

**Fig. 1.**
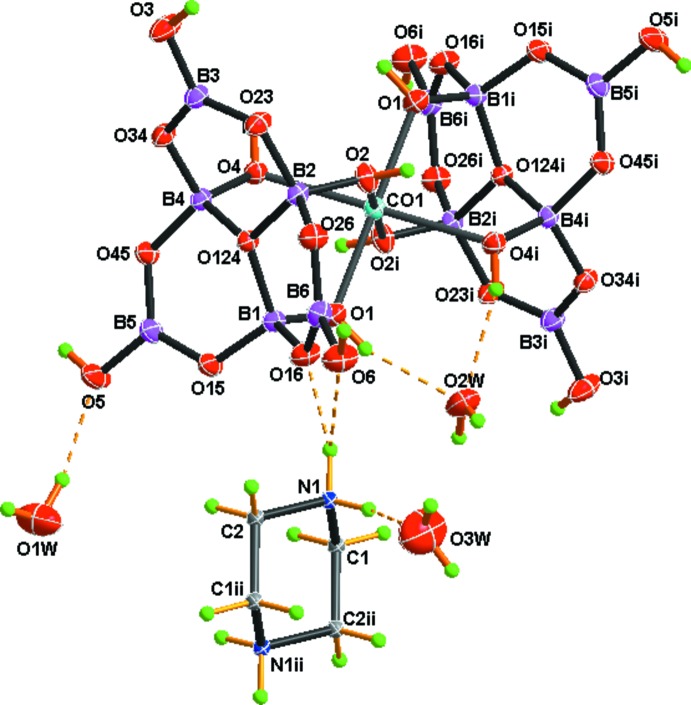
An ORTEP view of (I) with displacement ellipsoids drawn at the 30% probability level. Hydrogen bonds are represented as dashed lines. [Symmetry code: (i) 1 - x, 1 - y, 1 - z]

**Fig. 2.**
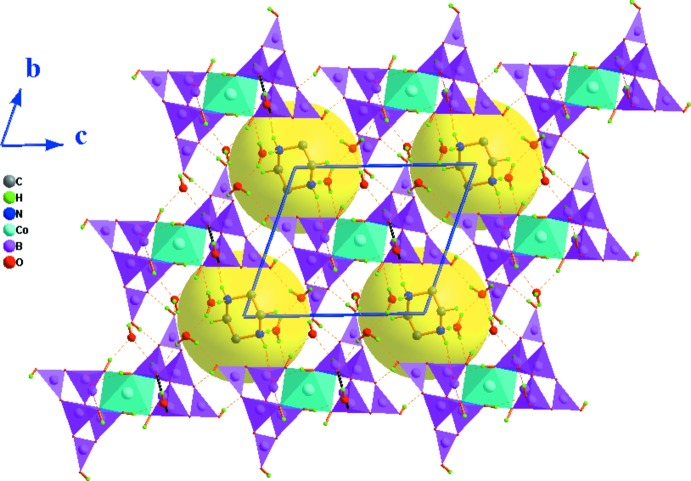
Crystal structure of I viewed along a axis showing a three-dimensional-supramolecular structure featuring the voids represented as large yellow ball. The H-atoms not included in H-bond scheme are omitted.

## supplementary crystallographic information

### 1. Comment

The mode of condensation of trigonal planar B(OH)_3_ and tetrahedral [B(OH)_4_]^-^ primary structural units, leads to a variety of hydrated polyborate structures such as rings, cages, chains, sheets or extended networks. In particular, the polymerization of three tetrahedral BO_3_(OH) (3Δ) and three trigonal BO2(OH)(3 T) moieties form tricyclic [B_6_O_7_(OH)_6_]^2-^ anions featuring an unusual three-coordinate oxygen atom (µ_3_–O). To date, various hydrated hexaborate structures including inorganic metals have been extensively studied. In contrast, those with organic and inorganic cations are less explored. On the best of our knowledge only one hydrated hexaborate with transition metal and organic cations, is earlier cited; [(Me)_2_NH(CH_2_)_2_NH(Me)_2_]{Zn[B_6_O_7_(OH)_6_]2}·2H_2_O (with Me = methyl)(Natarajan *et al.*, 2003). In this work, we report the synthesis and structure of a new organic-inorganic hydrated hexaborate, [C_4_N_2_H_12_]{Co[B_6_O_7_(OH)_6_]_2_}·6H_2_O (I).

The asymmetric unit of the title complex consists of a half [C_4_H_12_N_2_]^2+^ diprotonated cation, a half of {Co[B_6_O_7_(OH)_6_]_2_}^2-^ complex hexaborate anions and three lattice water molecules as shown in Figure 1. As the Co^2+^ ion is located on centre of inversion so is bonded to six O atoms of two chelating [B_6_O_7_(OH)_6_]^2-^ anions forming thus a distorted octahedron. The Co—O distances range from 2.0539 (17) to 2.1926 (12) Å and the O—Co—O angles are between 87.03 (5)–180.00 (7)° (Table 1). The [B_6_O_7_(OH)_6_]^2-^ unit is formed by three tetrahedral B and three trigonal B atoms (3Δ + 3 T), linked through six double bridged (µ_2_–O) and one triple bridged (µ_3_–O) oxygen atoms in which each B atom possesses one terminal OH group, resulting in the formation of a tricyclic honeycomb-like structure anion (Fig. 2). Similar hexaborate anions have been encountered in the mineral aksaite (Negro *et al.*, 1971).

The B—O and O—B—O bond lengths range from 1.434 (2) to 1.5123 (18) Å and from 105.58 (12) to 112.53 (12) ° respectively for the tetrahedral B atoms and those for trigonal ones are in the range 1.355 (2)–1.371 (2) Å and from 114.61 (14) to 122.88 (14) °. The longer B—O distances are observed for the tetrahedral B atoms associated with the µ_3_ oxygen atom. These structural parameters are in good agreement with those observed for other hydrated hexaborate associated with some alkali and transition metals, such as K_2_Mg[B_6_O_7_(OH)_6_]_2_·4H_2_O (Zhihong *et al.*, 2005) and Rb_2_Co[B_6_O_7_(OH)_6_]_2_·4H_2_O (Yue *et al.*, 2003).

The [B_6_O_7_(OH)_6_]^2-^ anions are further interconnected through O—H···O hydrogen-bonding interactions of the hydroxyl groups (Table 2) yelding to a three-dimensional framework with channels along the *a* axis. The negative excess charges of the anionic framework is compensated by [C_4_H_12_N_2_]^2+^ cations, which occupy the voids along with water molecules (Fig. 3). It'to be noted that the diprotonated piperazine ring adopts a chair conformation, with puckering parameters Q = 1.7803 Å, θ = 171.58° and φ = 90°.

### 2. Experimental

Piperazine (0.086 g), CoCl_2_·6H_2_O (0.8 g) and H_3_BO_3_ (2 g) in a molar ratio: piperazine/Co/ B = 1/4/32 were dissolved in a mixture of 30 ml of distilled water and 20 ml of ethanol and then stirred for 1 h. The pH was adjusted to 9 by addition of 5 ml of pyridine. Within two weeks, pink prisms of the title compound were obtained. The existence of hexaborate anion in the prepared compound is evidenced by characteristic IR absorption band at 685 cm^-1^. IR (KBr, cm^-1^): 1358 ν_as_(BO_3_), 908–850 ν_s_(BO_3_), 1028ν_as_(BO_4_),807 ν_s_(BO_4_),1095 ν(BOH) and 685δ(BO_3_)+δ(BO_4_).

### 3. Refinement

All H atoms attached to C and N atoms were fixed geometrically and treated as riding, with C—H = 0.97 Å and N—H = 0.90 Å with *U*_iso_(H) = 1.2Ueq(C, N) for piperazine ring. The H atoms of OH groups are fixed using restraint O—H = 0.82 Å and those of water molecules are located using restraints [O—H = 0.85 (1) Å, H···H = 1.44 (2) Å and *U*_iso_(H) = 1.5Ueq(O). Four bad reflections with (Iobs-Icalc)/SigmaW > 10 are omitted on the final refinement.

### Crystal data

**Table:**

(C_4_H_12_N_2_)[Co(H_6_B_6_O_13_)_2_]·6H_2_O	*Z* = 1
*M**_r_* = 813.00	*F*(000) = 417
Triclinic, *P*1	*D*_x_ = 1.696 Mg m^−^^3^
Hall symbol: -P 1	Ag *K*α radiation, λ = 0.56087 Å
*a* = 8.226 (3) Å	Cell parameters from 25 reflections
*b* = 10.157 (2) Å	θ = 9–11°
*c* = 11.298 (4) Å	µ = 0.35 mm^−^^1^
α = 65.98 (2)°	*T* = 298 K
β = 74.20 (3)°	Prism, pink
γ = 69.03 (5)°	0.23 × 0.15 × 0.11 mm
*V* = 796.2 (4) Å^3^	

### Data collection

**Table:**

Enraf–Nonius CAD-4 diffractometer	*R*_int_ = 0.014
Radiation source: fine-focus sealed tube	θ_max_ = 28.0°, θ_min_ = 2.1°
Graphite monochromator	*h* = −13→13
non–profiled ω scans	*k* = −16→16
9895 measured reflections	*l* = −3→18
7785 independent reflections	2 standard reflections every 120 min
6118 reflections with *I* > 2σ(*I*)	intensity decay: 2%

### Refinement

**Table:**

Refinement on *F*^2^	Primary atom site location: structure-invariant direct methods
Least-squares matrix: full	Secondary atom site location: difference Fourier map
*R*[*F*^2^ > 2σ(*F*^2^)] = 0.049	Hydrogen site location: inferred from neighbouring sites
*wR*(*F*^2^) = 0.153	H atoms treated by a mixture of independent and constrained refinement
*S* = 1.06	*w* = 1/[σ^2^(*F*_o_^2^) + (0.0924*P*)^2^ + 0.2147*P*] where *P* = (*F*_o_^2^ + 2*F*_c_^2^)/3
7785 reflections	(Δ/σ)_max_ < 0.001
259 parameters	Δρ_max_ = 2.42 e Å^−^^3^
12 restraints	Δρ_min_ = −0.99 e Å^−^^3^

### Special details

**Table:**

Geometry. All e.s.d.'s (except the e.s.d. in the dihedral angle between two l.s. planes) are estimated using the full covariance matrix. The cell e.s.d.'s are taken into account individually in the estimation of e.s.d.'s in distances, angles and torsion angles; correlations between e.s.d.'s in cell parameters are only used when they are defined by crystal symmetry. An approximate (isotropic) treatment of cell e.s.d.'s is used for estimating e.s.d.'s involving l.s. planes.
Refinement. Refinement of *F*^2^ against ALL reflections. The weighted *R*-factor *wR* and goodness of fit *S* are based on *F*^2^, conventional *R*-factors *R* are based on *F*, with *F* set to zero for negative *F*^2^. The threshold expression of *F*^2^ > σ(*F*^2^) is used only for calculating *R*-factors(gt) *etc*. and is not relevant to the choice of reflections for refinement. *R*-factors based on *F*^2^ are statistically about twice as large as those based on *F*, and *R*- factors based on ALL data will be even larger.

### Fractional atomic coordinates and isotropic or equivalent isotropic displacement parameters (Å^2^)

**Table:**

	*x*	*y*	*z*	*U*_iso_*/*U*_eq_	
Co1	0.5000	0.5000	0.5000	0.01614 (7)	
B1	0.7384 (2)	0.59885 (17)	0.23924 (15)	0.0163 (2)	
B2	0.8734 (2)	0.33738 (17)	0.39832 (16)	0.0167 (2)	
B3	0.8346 (2)	0.12594 (18)	0.36263 (17)	0.0200 (3)	
B4	0.6375 (2)	0.37212 (16)	0.26842 (15)	0.0161 (2)	
B5	0.6174 (2)	0.59847 (19)	0.06603 (16)	0.0210 (3)	
B6	1.0368 (2)	0.5217 (2)	0.29973 (17)	0.0203 (3)	
O124	0.77220 (13)	0.43050 (11)	0.28482 (10)	0.01562 (17)	
O45	0.58438 (15)	0.46319 (12)	0.14086 (11)	0.02026 (19)	
O34	0.71305 (15)	0.21657 (12)	0.28139 (12)	0.02037 (19)	
O16	0.89561 (14)	0.62883 (13)	0.24323 (12)	0.0225 (2)	
O15	0.69605 (17)	0.66434 (13)	0.10864 (11)	0.0232 (2)	
O23	0.91602 (16)	0.17965 (12)	0.41590 (12)	0.0229 (2)	
O1	0.58976 (15)	0.65398 (12)	0.33104 (12)	0.02076 (19)	
H1	0.595 (4)	0.7336 (18)	0.330 (3)	0.031*	
O2	0.75590 (15)	0.36962 (14)	0.51227 (11)	0.0222 (2)	
H2	0.793 (4)	0.361 (3)	0.5750 (18)	0.033*	
O3	0.8757 (2)	−0.02567 (13)	0.38981 (16)	0.0321 (3)	
H3	0.9463	−0.0701	0.4421	0.048*	
O4	0.51422 (14)	0.61177 (12)	0.62547 (11)	0.01811 (18)	
H4	0.528 (4)	0.6931 (17)	0.578 (2)	0.027*	
O5	0.5670 (2)	0.67326 (16)	−0.05561 (13)	0.0352 (3)	
H5	0.5205	0.6238	−0.0708	0.053*	
O6	1.17955 (16)	0.57172 (15)	0.27863 (15)	0.0286 (3)	
H6	1.2628	0.5003	0.3070	0.043*	
O26	1.03454 (14)	0.37793 (13)	0.37106 (12)	0.0223 (2)	
O1W	0.6861 (3)	0.8794 (2)	−0.2835 (2)	0.0489 (4)	
H1W1	0.622 (4)	0.833 (4)	−0.216 (3)	0.073*	
H2W1	0.773 (3)	0.818 (3)	−0.314 (3)	0.073*	
O2W	0.5784 (2)	0.87545 (16)	0.41668 (16)	0.0349 (3)	
H1W2	0.676 (2)	0.882 (3)	0.423 (3)	0.052*	
H2W2	0.511 (3)	0.959 (2)	0.375 (3)	0.052*	
O3W	1.2705 (3)	0.9010 (3)	0.2089 (3)	0.0675 (7)	
H2W3	1.253 (5)	0.981 (2)	0.223 (5)	0.101*	
H1W3	1.3578	0.8334	0.2430	0.101*	
N1	0.9898 (3)	0.8815 (2)	0.1223 (2)	0.0443 (4)	
H1A	0.9538	0.8001	0.1763	0.053*	
H1B	1.0545	0.8982	0.1646	0.053*	
C1	0.8360 (3)	1.0119 (2)	0.0893 (2)	0.0355 (4)	
H1C	0.7653	1.0290	0.1686	0.043*	
H1D	0.7638	0.9935	0.0458	0.043*	
C2	1.1021 (4)	0.8520 (2)	−0.0001 (3)	0.0441 (5)	
H2A	1.2028	0.7651	0.0235	0.053*	
H2B	1.0339	0.8314	−0.0450	0.053*	

### Atomic displacement parameters (Å^2^)

**Table:**

	*U*^11^	*U*^22^	*U*^33^	*U*^12^	*U*^13^	*U*^23^
Co1	0.01484 (11)	0.01703 (12)	0.01733 (12)	−0.00406 (8)	−0.00425 (8)	−0.00587 (9)
B1	0.0158 (5)	0.0161 (5)	0.0180 (6)	−0.0053 (4)	−0.0062 (5)	−0.0038 (5)
B2	0.0157 (5)	0.0159 (5)	0.0194 (6)	−0.0025 (4)	−0.0083 (5)	−0.0050 (5)
B3	0.0214 (6)	0.0157 (6)	0.0240 (7)	−0.0030 (5)	−0.0078 (5)	−0.0070 (5)
B4	0.0168 (5)	0.0154 (5)	0.0179 (6)	−0.0039 (4)	−0.0071 (5)	−0.0053 (5)
B5	0.0255 (7)	0.0211 (6)	0.0173 (6)	−0.0073 (5)	−0.0086 (5)	−0.0034 (5)
B6	0.0160 (6)	0.0245 (7)	0.0241 (7)	−0.0074 (5)	−0.0055 (5)	−0.0090 (6)
O124	0.0151 (4)	0.0150 (4)	0.0180 (4)	−0.0037 (3)	−0.0070 (3)	−0.0046 (3)
O45	0.0244 (5)	0.0208 (4)	0.0182 (4)	−0.0091 (4)	−0.0102 (4)	−0.0025 (4)
O34	0.0233 (5)	0.0158 (4)	0.0252 (5)	−0.0029 (3)	−0.0108 (4)	−0.0078 (4)
O16	0.0176 (4)	0.0203 (4)	0.0316 (6)	−0.0072 (4)	−0.0091 (4)	−0.0058 (4)
O15	0.0321 (6)	0.0206 (5)	0.0194 (5)	−0.0107 (4)	−0.0116 (4)	−0.0014 (4)
O23	0.0262 (5)	0.0148 (4)	0.0294 (5)	−0.0011 (4)	−0.0159 (4)	−0.0058 (4)
O1	0.0199 (4)	0.0178 (4)	0.0254 (5)	−0.0055 (3)	−0.0020 (4)	−0.0088 (4)
O2	0.0187 (4)	0.0287 (5)	0.0192 (5)	−0.0025 (4)	−0.0074 (4)	−0.0091 (4)
O3	0.0400 (7)	0.0156 (5)	0.0460 (8)	−0.0014 (4)	−0.0265 (6)	−0.0085 (5)
O4	0.0174 (4)	0.0182 (4)	0.0203 (4)	−0.0065 (3)	−0.0039 (3)	−0.0061 (3)
O5	0.0583 (9)	0.0321 (6)	0.0227 (6)	−0.0253 (6)	−0.0218 (6)	0.0042 (5)
O6	0.0177 (5)	0.0287 (6)	0.0403 (7)	−0.0093 (4)	−0.0100 (5)	−0.0069 (5)
O26	0.0156 (4)	0.0235 (5)	0.0282 (5)	−0.0055 (4)	−0.0087 (4)	−0.0059 (4)
O1W	0.0409 (9)	0.0369 (8)	0.0484 (10)	−0.0071 (7)	0.0027 (7)	−0.0038 (7)
O2W	0.0407 (7)	0.0262 (6)	0.0426 (8)	−0.0149 (5)	−0.0062 (6)	−0.0116 (6)
O3W	0.0612 (14)	0.0639 (14)	0.0861 (17)	−0.0129 (11)	−0.0395 (13)	−0.0203 (13)
N1	0.0550 (12)	0.0363 (9)	0.0406 (10)	−0.0197 (8)	−0.0131 (9)	−0.0026 (8)
C1	0.0392 (10)	0.0318 (8)	0.0291 (8)	−0.0099 (7)	0.0008 (7)	−0.0081 (7)
C2	0.0509 (13)	0.0232 (8)	0.0485 (12)	−0.0079 (8)	0.0024 (10)	−0.0111 (8)

### Geometric parameters (Å, º)

**Table:**

Co1—O2	2.0539 (17)	B6—O6	1.368 (2)
Co1—O2^i^	2.0539 (17)	B6—O16	1.369 (2)
Co1—O1	2.0648 (14)	O1—H1	0.820 (10)
Co1—O1^i^	2.0648 (14)	O2—H2	0.811 (10)
Co1—O4^i^	2.1926 (12)	O3—H3	0.8200
Co1—O4	2.1926 (12)	O4—B4^i^	1.490 (2)
B1—O15	1.434 (2)	O4—H4	0.810 (10)
B1—O16	1.4462 (19)	O5—H5	0.8200
B1—O1	1.475 (2)	O6—H6	0.8200
B1—O124	1.5123 (18)	O1W—H1W1	0.861 (10)
B2—O26	1.4447 (19)	O1W—H2W1	0.856 (10)
B2—O23	1.4510 (19)	O2W—H1W2	0.852 (10)
B2—O2	1.462 (2)	O2W—H2W2	0.854 (10)
B2—O124	1.5041 (19)	O3W—H2W3	0.846 (10)
B3—O34	1.357 (2)	O3W—H1W3	0.847 (2)
B3—O23	1.363 (2)	N1—C1	1.466 (3)
B3—O3	1.371 (2)	N1—C2	1.517 (4)
B4—O34	1.4360 (19)	N1—H1A	0.9000
B4—O45	1.4463 (19)	N1—H1B	0.9000
B4—O4^i^	1.490 (2)	C1—C2^ii^	1.508 (3)
B4—O124	1.5117 (18)	C1—H1C	0.9700
B5—O15	1.356 (2)	C1—H1D	0.9700
B5—O5	1.368 (2)	C2—C1^ii^	1.508 (3)
B5—O45	1.368 (2)	C2—H2A	0.9700
B6—O26	1.355 (2)	C2—H2B	0.9700
			
O2—Co1—O2^i^	180.0	B2—O124—B4	118.01 (11)
O2—Co1—O1	88.68 (6)	B2—O124—B1	116.87 (11)
O2^i^—Co1—O1	91.32 (6)	B4—O124—B1	117.76 (11)
O2—Co1—O1^i^	91.32 (6)	B5—O45—B4	124.30 (12)
O2^i^—Co1—O1^i^	88.68 (6)	B3—O34—B4	120.52 (12)
O1—Co1—O1^i^	180.0	B6—O16—B1	124.47 (12)
O2—Co1—O4^i^	88.17 (5)	B5—O15—B1	120.85 (12)
O2^i^—Co1—O4^i^	91.83 (5)	B3—O23—B2	123.44 (12)
O1—Co1—O4^i^	87.03 (5)	B1—O1—Co1	118.94 (9)
O1^i^—Co1—O4^i^	92.97 (5)	B1—O1—H1	109.3 (19)
O2—Co1—O4	91.83 (5)	Co1—O1—H1	121.9 (19)
O2^i^—Co1—O4	88.17 (5)	B2—O2—Co1	121.28 (9)
O1—Co1—O4	92.97 (5)	B2—O2—H2	122 (2)
O1^i^—Co1—O4	87.03 (5)	Co1—O2—H2	114 (2)
O4^i^—Co1—O4	180.0	B3—O3—H3	109.5
O15—B1—O16	110.89 (12)	B4^i^—O4—Co1	116.22 (9)
O15—B1—O1	110.91 (13)	B4^i^—O4—H4	110.6 (19)
O16—B1—O1	110.56 (12)	Co1—O4—H4	107.3 (19)
O15—B1—O124	109.07 (12)	B5—O5—H5	109.5
O16—B1—O124	108.17 (12)	B6—O6—H6	109.5
O1—B1—O124	107.12 (12)	B6—O26—B2	119.33 (13)
O26—B2—O23	109.16 (13)	H1W1—O1W—H2W1	112 (2)
O26—B2—O2	112.53 (12)	H1W2—O2W—H2W2	113 (2)
O23—B2—O2	110.85 (13)	H2W3—O3W—H1W3	111 (2)
O26—B2—O124	109.35 (12)	C1—N1—C2	110.90 (19)
O23—B2—O124	109.28 (12)	C1—N1—H1A	109.5
O2—B2—O124	105.58 (12)	C2—N1—H1A	109.5
O34—B3—O23	122.88 (14)	C1—N1—H1B	109.5
O34—B3—O3	117.27 (14)	C2—N1—H1B	109.5
O23—B3—O3	119.85 (14)	H1A—N1—H1B	108.0
O34—B4—O45	111.78 (12)	N1—C1—C2^ii^	109.0 (2)
O34—B4—O4^i^	109.79 (12)	N1—C1—H1C	109.9
O45—B4—O4^i^	110.85 (12)	C2^ii^—C1—H1C	109.9
O34—B4—O124	109.48 (12)	N1—C1—H1D	109.9
O45—B4—O124	107.67 (11)	C2^ii^—C1—H1D	109.9
O4^i^—B4—O124	107.13 (11)	H1C—C1—H1D	108.3
O15—B5—O5	117.83 (14)	C1^ii^—C2—N1	109.18 (18)
O15—B5—O45	122.48 (14)	C1^ii^—C2—H2A	109.8
O5—B5—O45	119.68 (14)	N1—C2—H2A	109.8
O26—B6—O6	122.85 (15)	C1^ii^—C2—H2B	109.8
O26—B6—O16	122.54 (14)	N1—C2—H2B	109.8
O6—B6—O16	114.61 (14)	H2A—C2—H2B	108.3
			
O26—B2—O124—B4	158.42 (12)	O1—B1—O15—B5	−88.84 (17)
O23—B2—O124—B4	38.99 (17)	O124—B1—O15—B5	28.90 (19)
O2—B2—O124—B4	−80.28 (14)	O34—B3—O23—B2	4.1 (3)
O26—B2—O124—B1	−52.13 (16)	O3—B3—O23—B2	−176.41 (15)
O23—B2—O124—B1	−171.56 (11)	O26—B2—O23—B3	−136.34 (15)
O2—B2—O124—B1	69.17 (15)	O2—B2—O23—B3	99.17 (17)
O34—B4—O124—B2	−47.01 (16)	O124—B2—O23—B3	−16.8 (2)
O45—B4—O124—B2	−168.73 (11)	O15—B1—O1—Co1	128.21 (10)
O4^i^—B4—O124—B2	71.99 (15)	O16—B1—O1—Co1	−108.36 (12)
O34—B4—O124—B1	163.82 (12)	O124—B1—O1—Co1	9.28 (14)
O45—B4—O124—B1	42.10 (16)	O2—Co1—O1—B1	37.04 (10)
O4^i^—B4—O124—B1	−77.18 (14)	O2^i^—Co1—O1—B1	−142.96 (10)
O15—B1—O124—B2	160.84 (12)	O1^i^—Co1—O1—B1	3 (100)
O16—B1—O124—B2	40.14 (16)	O4^i^—Co1—O1—B1	−51.19 (10)
O1—B1—O124—B2	−79.05 (14)	O4—Co1—O1—B1	128.81 (10)
O15—B1—O124—B4	−49.63 (16)	O26—B2—O2—Co1	125.40 (11)
O16—B1—O124—B4	−170.33 (12)	O23—B2—O2—Co1	−112.03 (12)
O1—B1—O124—B4	70.47 (15)	O124—B2—O2—Co1	6.19 (15)
O15—B5—O45—B4	−3.7 (3)	O2^i^—Co1—O2—B2	−124 (100)
O5—B5—O45—B4	177.61 (16)	O1—Co1—O2—B2	−47.25 (11)
O34—B4—O45—B5	−135.19 (15)	O1^i^—Co1—O2—B2	132.75 (11)
O4^i^—B4—O45—B5	101.96 (17)	O4^i^—Co1—O2—B2	39.82 (11)
O124—B4—O45—B5	−14.9 (2)	O4—Co1—O2—B2	−140.18 (11)
O23—B3—O34—B4	−12.8 (2)	O2—Co1—O4—B4^i^	−134.31 (9)
O3—B3—O34—B4	167.68 (15)	O2^i^—Co1—O4—B4^i^	45.69 (9)
O45—B4—O34—B3	151.66 (14)	O1—Co1—O4—B4^i^	136.92 (10)
O4^i^—B4—O34—B3	−84.90 (16)	O1^i^—Co1—O4—B4^i^	−43.08 (10)
O124—B4—O34—B3	32.44 (19)	O4^i^—Co1—O4—B4^i^	10 (100)
O26—B6—O16—B1	−7.6 (3)	O6—B6—O26—B2	174.61 (15)
O6—B6—O16—B1	172.53 (15)	O16—B6—O26—B2	−5.2 (2)
O15—B1—O16—B6	−129.57 (16)	O23—B2—O26—B6	152.56 (14)
O1—B1—O16—B6	106.99 (17)	O2—B2—O26—B6	−83.94 (17)
O124—B1—O16—B6	−10.0 (2)	O124—B2—O26—B6	33.05 (19)
O5—B5—O15—B1	174.55 (16)	C2—N1—C1—C2^ii^	−59.8 (3)
O45—B5—O15—B1	−4.2 (3)	C1—N1—C2—C1^ii^	59.9 (3)
O16—B1—O15—B5	147.91 (15)		

Symmetry codes: (i) −*x*+1, −*y*+1, −*z*+1; (ii) −*x*+2, −*y*+2, −*z*.

### Hydrogen-bond geometry (Å, º)

**Table:**

*D*—H···*A*	*D*—H	H···*A*	*D*···*A*	*D*—H···*A*
O1—H1···O2*W*	0.82 (1)	2.00 (2)	2.7525 (19)	153 (3)
O2—H2···O6^iii^	0.81 (1)	2.11 (1)	2.876 (2)	157 (3)
O3—H3···O23^iv^	0.82	1.93	2.745 (2)	172
O4—H4···O2*W*	0.81 (1)	2.08 (1)	2.864 (2)	164 (3)
O5—H5···O45^v^	0.82	1.91	2.7159 (18)	169
O6—H6···O4^iii^	0.82	1.95	2.734 (2)	160
O1*W*—H1*W*1···O5	0.86 (1)	1.96 (2)	2.771 (2)	158 (4)
O1*W*—H2*W*1···O26^vi^	0.86 (1)	2.27 (1)	3.108 (3)	167 (3)
O1*W*—H2*W*1···O23^vi^	0.86 (1)	2.51 (3)	3.158 (3)	133 (3)
O2*W*—H1*W*2···O3^vii^	0.85 (1)	2.05 (2)	2.861 (2)	159 (3)
O2*W*—H2*W*2···O1*W*^viii^	0.85 (1)	2.01 (1)	2.838 (3)	164 (3)
O3*W*—H2*W*3···O1*W*^ii^	0.85 (1)	2.05 (2)	2.842 (3)	155 (3)
O3*W*—H1*W*3···O1^ix^	0.85 (1)	2.24 (1)	3.088 (3)	179 (1)
N1—H1*A*···O16	0.90	1.78	2.654 (2)	165
N1—H1*A*···O6	0.90	2.46	2.986 (3)	117
N1—H1*B*···O3*W*	0.90	1.98	2.836 (3)	157
C1—H1*C*···O34^vii^	0.97	2.56	3.321 (3)	136

Symmetry codes: (ii) −*x*+2, −*y*+2, −*z*; (iii) −*x*+2, −*y*+1, −*z*+1; (iv) −*x*+2, −*y*, −*z*+1; (v) −*x*+1, −*y*+1, −*z*; (vi) −*x*+2, −*y*+1, −*z*; (vii) *x*, *y*+1, *z*; (viii) −*x*+1, −*y*+2, −*z*; (ix) *x*+1, *y*, *z*.
